# Catecholamine-Induced Cardiomyopathy Secondary to Pheochromocytoma Rescued With Percutaneous Left Ventricular Assist Device: Novel Application of the Impella CP in a Pediatric Patient

**DOI:** 10.31486/toj.24.0028

**Published:** 2024

**Authors:** Christopher M. Zumwalt, Jessica Roybal, Ivory Crittendon, Victor S. Lucas, Dennis A. Wells

**Affiliations:** ^1^ Department of Surgery, Ochsner Clinic Foundation, New Orleans, LA; ^2^ Division of Pediatric General Surgery, Ochsner Children's Hospital, New Orleans, LA; ^3^ Division of Pediatric Cardiology, Ochsner Children's Hospital, New Orleans, LA; ^4^ Division of Pediatric and Congenital Cardiac Surgery, Ochsner Children's Hospital, New Orleans, LA

**Keywords:** *Cardiomyopathies*, *catecholamines*, *heart-assist devices*, *pediatrics*, *pheochromocytoma*

## Abstract

**Background:** Catecholamine-induced cardiomyopathy is an uncommon complication of pheochromocytoma. Pheochromocytoma is a rare tumor that predominantly occurs in adults, making catecholamine-induced cardiomyopathy secondary to pheochromocytoma in children an exceedingly rare presentation. Treatment typically consists of medical management followed by surgical resection. Mechanical support, typically salvage therapy with extracorporeal membrane oxygenation, has been used in adult patients with cardiogenic shock and after cardiac arrest, but to our knowledge, the use of mechanical support has not been described in pediatric patients.

**Case Report:** A 16-year-old female presented with cardiogenic shock resulting from catecholamine-induced cardiomyopathy secondary to pheochromocytoma. She was treated with a percutaneous left ventricular assist device to allow myocardial recovery while medical therapy was optimized. Given the early initiation, the patient's myocardial recovery was prompt, and only 3 days of device support were required. She was discharged in good condition and subsequently underwent uncomplicated laparoscopic resection of the tumor a few weeks later.

**Conclusion:** In pediatric patients with catecholamine-induced cardiomyopathy secondary to pheochromocytoma, aggressive measures of support—including mechanical support and infrequently used options such as percutaneous left ventricular assist devices—should be considered early in treatment to maintain adequate cardiac output, avoid cardiac arrest, and allow for prompt myocardial recovery.

## INTRODUCTION

Pheochromocytomas are catecholamine-secreting neuroendocrine tumors that occur in the adrenal gland. The incidence of pheochromocytoma is rare, and estimates are often combined with the paraganglioma, a similar tumor that occurs anywhere along the sympathetic and parasympathetic paraganglia. Together, the incidence of these tumors is estimated at <0.3 cases per million per year.^1^ The majority of these rare tumors occur in adults, with only 10% to 20% diagnosed in pediatric patients.^1^

Catecholamine-induced cardiomyopathy is a rare complication of pheochromocytoma, occurring in only 8% to 11% of all cases, and is seldom a presenting symptom.^2^ Treatment typically consists of medical management followed by surgical resection. The mainstay of medical management is supportive care and administration of an alpha-blocker to control blood pressure for at least a week (and often more) prior to surgery, with usually the subsequent addition of a beta-blocker and other agents in the interim prior to surgery.^2^ Mechanical support has been used in adult patients who develop cardiogenic shock. The type of support in such cases has been extracorporeal membrane oxygenation (ECMO), often used as salvage therapy after complications of shock have occurred or after cardiac arrest.^3-5^

Given the rarity of catecholamine-induced cardiomyopathy secondary to pheochromocytoma and the rarity of these tumors among pediatric patients, the literature about this condition in pediatric patients is limited. We were able to identify only 1 case reporting a pediatric patient with catecholamine-induced cardiomyopathy as a complication of pheochromocytoma.^6^ However, the patient's ventricular function was only mildly reduced, and cardiogenic shock was not present.^6^ We are unaware of any reports of successful use of mechanical support to treat a pediatric patient with catecholamine-induced cardiomyopathy secondary to pheochromocytoma. To our knowledge, our case is the first published report of cardiogenic shock in a pediatric patient with pheochromocytoma and the first use of a percutaneous left ventricular assist device in such a case.

## CASE REPORT

A 16-year-old otherwise healthy female with a medical history significant only for mild intermittent asthma presented to an outside facility after developing sudden chest pain while playing basketball at school. On arrival, she was hypertensive (blood pressure of 210/150 mm Hg) and in sinus tachycardia (heart rate up to 115 beats per minute). Laboratory values were remarkable only for elevated troponin and brain natriuretic peptide, 12.19 ng/mL (reference value, <0.04 ng/mL) and 507 pg/mL (reference value, <100 pg/mL), respectively. Computed tomography angiography was negative for pulmonary embolism.

The patient developed progressive respiratory failure and was transferred to the pediatric intensive care unit (PICU) where she was intubated and placed on mechanical ventilation support. After intubation, she had persistent tachycardia (heart rate up to 190 beats per minute), but her blood pressure was reported to be only mildly elevated at that point (systolic blood pressure of 140 to 150 mm Hg). Echocardiogram demonstrated a left ventricular ejection fraction <20%.

Of note, the patient had had no recent significant respiratory or other viral symptoms, and a viral panel and urine toxicology screen were negative. Her family history was negative for cardiomyopathies, and later genetic testing was also negative. She was started on milrinone at 0.3 μg/kg/min and transferred from Baton Rouge, Louisiana, to the Ochsner Children's Hospital PICU in New Orleans, Louisiana, for evaluation for advanced heart failure therapies.

On arrival in the afternoon at our facility, she continued to be tachycardic (heart rate in the range of 170 to the 180s beats per minute) and hypertensive (systolic blood pressure 130 to 140 mm Hg), so sodium nitroprusside 0.7 μg/kg/min was added for afterload control. Repeat echocardiogram continued to demonstrate severe left ventricular dysfunction with fractional shortening of 7.0% (reference range, 28.9%-42.5%).

The heart failure team, including a congenital cardiac surgeon, cardiologists, and intensivists, discussed options for support and potential etiologies. Given the degree of tachycardia and the patient's hypertension, the possibility of pheochromocytoma was discussed, and confirmatory diagnostics were planned. However, the patient was in cardiogenic shock with acute risk for decompensation. She was an appropriate size for percutaneous ventricular assist device therapy in the form of the Impella CP (Abiomed, Inc). A ventricular assist device was felt to be superior to and lower risk than ECMO given that only left ventricular support was necessary. The patient's right ventricular function was adequate, and oxygenation and lung compliance were adequate with mechanical ventilation. The Impella CP device would offer better unloading of the left ventricle than medical therapy alone. A plan was made to place the device if any evidence of worsening was seen while medical therapy was being optimized. Oliguria developed within a few hours of the patient's arrival, triggering the team to proceed with insertion of the Impella CP that evening.

The patient was taken to the catheterization laboratory with pediatric interventional cardiologists and the congenital cardiac surgeon. Percutaneous femoral arterial access was planned if the femoral vessels were large enough to accommodate the device with low risk of impaired limb perfusion. The team was prepared to sew a vascular graft onto the femoral artery and insert the device through the graft if necessary to prevent limb ischemia. However, the femoral vessels were large enough to accommodate the device without requiring a graft, and a persistent sciatic artery provided additional protection to the distal limb perfusion. The insertion was uncomplicated, and the device provided excellent support to the patient's cardiac output. Urine output improved immediately after initiation of support, the patient was quickly weaned from the milrinone and sodium nitroprusside, and her fractional shortening increased to 20% by postoperative day 1 and to 22% on postoperative day 2. Coronary angiography performed at the time was unremarkable.

Renal ultrasound subsequently identified an adrenal mass, and magnetic resonance imaging confirmed the presence of pheochromocytoma. Alpha blockade was initiated with doxazosin 10 mg daily. Urine catecholamines were normal; however, plasma metanephrines were markedly elevated at 17,906 pg/mL (reference value, <205 pg/mL).

The patient's ventricular recovery was prompt after optimization of medical therapy. Two days after insertion of the Impella CP, a weaning trial of the device demonstrated only mildly reduced left ventricular function with fractional shortening of 22%, so removal was planned. Removal was performed in the catheterization laboratory with a percutaneous closure device and was complicated by femoral artery dissection that was immediately recognized and repaired by vascular surgery with a good result and no complications to the affected extremity.

The patient was extubated the following day after 3 days of mechanical ventilation and was moved from the PICU to the stepdown unit on hospital day 6. She continued to recover well on the floor and was discharged with normal biventricular function after 12 days in the hospital. She was discharged on doxazosin 10 mg daily and atenolol 12.5 mg daily for blood pressure and heart rate control. Adrenalectomy was planned for a few weeks later.

The patient underwent laparoscopic right adrenalectomy 6 weeks later. The case was uncomplicated, and she was discharged on postoperative day 2. Pathology reported a 4.5-cm pheochromocytoma negative for malignancy. The patient continues to do well with normal biventricular function 7 months after discharge.

## DISCUSSION

Pheochromocytoma complicated by catecholamine-induced cardiomyopathy and cardiogenic shock is an incredibly rare presentation, especially in pediatric patients. Hemodynamic stabilization is the goal of initial pheochromocytoma management; if the initial presentation is confounded by cardiogenic shock, as in our patient, temporary mechanical circulatory support may be necessary.

We suggest that mechanical support—including the novel application of less invasive, percutaneous devices rather than implanted devices—should be considered early in the management of appropriately sized pediatric patients to avoid cardiac arrest and to facilitate a less-complicated and quicker recovery. The left ventricular unloading specifically provided by devices such as the Impella CP or Impella 5.5 (Abiomed, Inc) seems ideally suited for treatment of catecholamine-induced cardiomyopathy resulting from pheochromocytoma. Reports of these devices used as support in adults with this diagnosis, often in combination with ECMO or after cardiac arrest, have been published,^7-10^ but at least 2 recent (2021, 2022) cases report the devices were used as the sole form of medical support in an adult and prior to cardiac arrest.^9,10^ Left ventricular assist devices have been used infrequently in pediatric patients for any form of heart failure, but we have used them successfully in a number of cases in appropriately sized patients. To our knowledge, the case we present here is the first successful application of the Impella CP for the treatment of catecholamine-induced cardiomyopathy secondary to pheochromocytoma in a pediatric case.

This unusual case highlights the importance of comprehensive multidisciplinary care and teamwork in the care of complex patients, rare presentations, and advanced heart failure. Regardless of the etiology of the heart failure, early aggressive support generally serves the patient better than a reactionary approach to multiorgan failure or cardiac arrest. When a presentation is acute and the etiology is unclear, delays in recognition of shock and in implementation of advanced therapies can increase the risk of a bad outcome. However, the types of mechanical support devices in the armamentarium at many pediatric centers is quite limited. Because most of the less invasive and percutaneous device options are designed for adults, appropriateness of size and fit limit the frequent use of such therapies in pediatric patients with advanced heart failure and cardiogenic shock. Use of Impella devices is limited by both ventricular chamber size and vessel size for access. However, in appropriately sized patients, early use may prevent multiorgan injury and increase the chance of recovery for catecholamine-induced cardiomyopathy specifically. In many such cases, the unloading of the left ventricle provided by Impella devices may be a better support strategy than ECMO, assuming ECMO is not necessary for concomitant lung disease or severe biventricular failure. We feel that early use of the Impella device contributed to the prompt myocardial recovery in our patient. While no form of mechanical support is without inherent risks, the hemodynamic support provided while the diagnosis was being confirmed and medical therapy was being optimized likely prevented further end organ damage and allowed for a more rapid and smooth recovery overall.

## CONCLUSION

Catecholamine-induced cardiomyopathy secondary to pheochromocytoma in pediatric patients is exceedingly rare, but aggressive measures such as mechanical support, including infrequently used options such as percutaneous left ventricular assist devices, should be considered early in treatment to maintain adequate cardiac output, avoid complications, and allow for a more prompt recovery. To our knowledge, this case is the first report of Impella CP device use in the pediatric population as a bridge to recovery in this setting and provides an example of how pediatric patients can be supported with such devices, even for novel indications. We hope this report stimulates interest among pediatric advanced heart failure teams and they keep this application in mind to optimize patient support and recovery.

## Notes

*This article meets the Accreditation Council for Graduate Medical Education and the American Board of Medical Specialties Maintenance of Certification competencies for Patient Care and Medical Knowledge.*
